# Identifying the factors affecting strategic decision-making ability to boost the entrepreneurial performance: A hybrid structural equation modeling – artificial neural network approach

**DOI:** 10.3389/fpsyg.2022.1038604

**Published:** 2022-10-31

**Authors:** Jiaying Feng, Ping Han, Wei Zheng, Asif Kamran

**Affiliations:** ^1^School of Economics and Management, Harbin University, Harbin, China; ^2^School of Economics, Harbin University of Commerce, Harbin, China; ^3^Department of Finance, Harbin University, Harbin, China; ^4^Faculty of Business Management and Study, Nazeer Hussain University, Karachi, Pakistan

**Keywords:** strategic decision-making, entrepreneurship, performance, cognitive, SEM

## Abstract

This study builds a conceptual model of strategic decision-making ability that leads to entrepreneurial performance (EP) based on the two-system decision-making theory and logical analysis. An empirical approach using structural equation modeling – artificial neural network (SEM-ANN) was performed to describe the linear and nonlinear relationships in the proposed model. The empirical results reveal that strategic decision-making abilities are affected by five factors: attention, memory, thinking, emotion, and sentiment, and whose influence mechanisms and degrees are varied. Results also describe that these abilities have a positive effect on overall EP. Therefore, results suggest that businesses’ strategic decision-making is usually strengthened when entrepreneurs have a clear understanding of these influencing elements, and the interaction between them leads to improved performance.

## Introduction

Entrepreneurship is thought to be a means of performance and wealth development. With the information economy expanding so quickly, knowledge-based decision-making is seen as a key tool for success and prosperity (Yang et al., 2018; Jiatong et al., 2021a). The key factor influencing the performance and profitability of organizations is strategic decision-making (Feng et al., 2022). Because, the willingness to take risks and make decisions, as well as organizational product invention and market innovation, are all linked to an individual’s entrepreneurial performance (EP) (Li et al., 2020a). Furthermore, there is a correlation between value creation and creative thinking, recognition of opportunities, as well as time, resources, risk, and other components sof strategic decision-making and EP (Theriou and Chatzoudes, 2015; Li et al., 2020a). In the current green environmental pressure, businesses must prospectively include competitive issues into their strategic plans to build innovative initiatives and achieve a foothold in the extremely competitive business world (Abbas, 2020). Research showed that managers’ decision-making skill affects the organization’s innovative strategies. It is indeed important to research what affects and improves managers’ decision-making ability (Pan et al., 2020).

Since the 21st century began, organizational culture has integrated IT and industries (Shahzad et al., 2017; Agarwala and Chaudhary, 2021). Strategic decisions to engage in innovation are significantly influenced by global technological growth, which produces new patterns of doing business (Xu et al., 2022). To increase management decision-making ability, enterprises should optimize the governance system, raise development awareness, design development plans based on long-term sustainable growth, and encourage enterprise strategy execution (Feng et al., 2022; Hu et al., 2022; Wang and Liu, 2022). Furthermore, strategic decision-making behavior requires organizations to match the external environment and internal resource capabilities; however, most are constrained by internal resources and are unable to develop flexible strategies (Li et al., 2020b). Several studies use cognitive-behavioral theory to evaluate cognitive behavior, claiming that it is critical to organizational decision-making, especially in the fields of entrepreneurship, technology adoption decisions, social media, and consumer behavior (Dwivedi et al., 2017; Chou et al., 2020; Wang et al., 2022). Strategic decision-making has not been linked to EP in previous research. Therefore, to fill a research void in organizational psychology, this study examines the factors that influence an entrepreneur’s abilities to make strategic decisions that ultimately lead to enhanced EP.

In a new current theory, behavioral change is used as a proxy for success or failure, and cognitive and affective aspects can be investigated as predictors of decision-making quality (Feng et al., 2022). The level of cognitive acuity not only allows for more information sharing but also reduces the stress of controversy (Kang and Lee, 2017). In a similar vein, as the importance of being able to make strategic decisions as an entrepreneur has increased, the study of such talents has attracted a significant amount of interest in both academic and business communities (Bilancini et al., 2019). The link between influencing factors and the ability to make strategic decisions is complex because strategic decision-making involves a cognitive and psychological transformation process (Narayanan et al., 2011). However, because the model is excessively complex, parameters are difficult to estimate, a huge amount of work is required, and the model is unstable, it is difficult to implement in practice (Kaplan, 2011). Therefore, it is also essential to determine whether the main factors that influence strategic decision-making are linear or non-linear in their interaction with one another to steer the EP. The research aims to establish a linear and nonlinear model between a particular aspect and strategic decision-making ability. SEM and ANN are used to express nonlinear relationships between variables and have excellent self-learning abilities. Academics have given little attention to research structure due to its complexity. Lack of research hinders the establishment of a thorough research system to determine the impact on strategic decision-making.

The performance of entrepreneurial enterprises is the output of entrepreneurial activities at the organizational level and an important embodiment of entrepreneurial success. Although entrepreneurs have unique entrepreneurial advantages, their EP is uneven in the face of the dual uncertainties of the market and technology. The environment of an enterprise is characterized by dynamic, uncertain, and complex characteristics, rather than being in a static and stable state. Enterprises need to realize adaptability between their resources and their pursued opportunities according to their environment and develop a strategic plan that can best match external environmental opportunities with internal resources. Adaptation of a strategy is a process of “matching” or “matching the organizational resources” with the opportunities of the environment (Ruobing et al., 2022). At present, there is a lack of relevant reasons in the academic circle, and only the difference in creativity characteristics during entrepreneurship is regarded as the influencing factor (Jiatong et al., 2021b; Xie et al., 2021), but the creativity characteristics are more the external expression of technical ability and transfer of knowledge (Zhou et al., 2021), which do not reflect the personal characteristics that affect the major decision-making and management of enterprises. Therefore, the key research questions of this study are framed as (1) What are the cognitive and affective factors that affect strategic decision-making abilities? (2) What is the impact of strategic decision-making abilities on EP? This research conclusion can be filling the research gap in organizational psychology, and provide countermeasures and suggestions for the improvement of enterprise EP.

## Literature review and hypotheses development

Enterprise strategic decision-making ability combines many ability factors. The research on strategic decision-making ability focuses on the composition of strategic decision-making ability and the factors that influence it (Wally and Baum, 1994; Bilancini et al., 2019). According to the literature, strategic decision-making ability encompasses three abilities: first, the capacity to locate, predict and capture strategic opportunities by assessing the environment. Second, is the ability to make strategic decisions such as setting a goal and deciding on a business strategy. The third step is to integrate resources by selecting, acquiring, and utilizing resources (Milliken and Vollrath, 1991; Wally and Baum, 1994). The study by Schønning et al. (2019) believes the strategic decision-making ability system has three dimensions: strategy analysis, strategy selection, and optimization, and adaptive and updating capacity. All elements work together to develop and perfect strategic decision-making. By carefully studying rational decision-making variables, a comprehensive and workable driving model is created (Spanuth et al., 2020).

Furthermore, enhancing a person’s capacity for strategic decision-making allows one to better understand both the environment’s special traits and the ever-changing trend (Bilancini et al., 2019). Strategic decision-making, especially in the realm of environmental sustainability, is the focus of academic research (Bilancini et al., 2019). Since demographic characteristics are mostly related to human capital and theoretical explanations are limited, scholars have begun to study the impact of entrepreneurial and executive team characteristics on the quality of strategic decision-making (Friedman and Carmeli, 2018; Feng et al., 2022). There is evidence from prior research suggesting a link between the aggression, core self-evaluation, and strategic decision-making abilities of the senior management team (Clohessy and Acton, 2019; Gao et al., 2021).

Recently, there has been intense interest in how the psychological makeup of decision-makers affects their ability to make sound strategic choices. The cognitive approach, complexity, requirements, and variation are the primary intellectual considerations (Forbes, 2007; Nadkarni and Narayanan, 2007). When seen from a cognitive viewpoint, the strategic decision-making process consists of three stages: environment scanning, interpretation, and action (Kumbure et al., 2020). In this research, we break the capability of making strategic decisions down into three categories: scanning, interpretation, and action ability. The capability to scan the environment for crucial information. The aptitude to make insightful and original sense of the data one has gathered. The capacity for logical appraisal and judgment, followed by the selection of appropriate actions in response to the environment, is what we mean when we talk of behavioral ability (Li et al., 2019).

Both an analytical, rational decision-making system and a heuristic, empirical system have been advocated in decision-making circles (Bryant, 2007; Shukla et al., 2019). The former is slow because it needs your brain to do the heavy lifting of applying logic rules and calculating probabilities. Cognition also plays a significant role in this process, as it is a form of decision-making. The latter make snappier judgments because of less deliberate engagement and the use of prior experience or logical associations. It follows that the ability of strategic decision-making is constrained not only by the cognitive aspects of decision-makers but also by cannot to resist the influence of emotional variables (Shahzad et al., 2022).

### Cognitive factors

The term “cognitive ability” is used to describe a person’s innate and fundamental intellectual function. This includes the person’s capacity for learning, reasoning, and communication (Chou et al., 2020). In theory, one’s logical prowess influences their decision to go out on their own. Furthermore, co-creation processes will aid in the development of better decisions (Anugrah and Hermawan, 2019). Individuals with higher cognitive abilities may foresee market conditions and trends more accurately, and they are frequently able to respond to rapidly changing markets in a timely and suitable manner (Bilancini et al., 2019). Attention, memory, and ideation are the most important cognitive factors. Through the selection and understanding of the development trend of things from a strategy perspective, a decision-maker with strong attention can receive rich information while ensuring objectivity and comprehensiveness of information (Brosch et al., 2013). A keen eye can also detect the drawbacks of strategic decision-making in real time and alter and change it in response to information input from its implementation (Jugovac and Cavallero, 2012). Simultaneously, attentiveness can assist in capturing the best chance for strategic judgments and ensuring the best return from strategic decisions (Sabet et al., 2017). Therefore, the following hypotheses are proposed in this research:

H1a: Attention has a positive effect on scanning ability.

H1b: Attention has a positive effect on interpretation ability.

H1c: Attention has a positive effect on action ability.

Memory is the ability to remember things. Memory’s accuracy and persistence provide quick, quality decisions. Before conveying the input to the target language audience, it must be kept in long-term memory in the source language (Yenkimaleki and van Heuven, 2017). Memory can store information and conclusions. When facing similar issues, it might leverage previous knowledge (Stocco et al., 2018). Strategic decision-making is memory-based. Strong-memory strategic decision-makers can accurately repeat valuable knowledge, enhancing decision-making efficiency and degree (Fechner et al., 2016). The large and deep network of information may help or traumatize employees’ sustainable innovation performance (Wang et al., 2018; Wiseman et al., 2022), which becomes part of their memory. A good memory can provide strategic decision-making concepts, processes, approaches, insights, and lessons (Bechara and Martin, 2004). Therefore, the following hypotheses are proposed in this research:

H2a: Memory has a positive effect on scanning ability.

H2b: Memory has a positive effect on interpretation ability.

H2c: Memory has a positive effect on action ability.

Ideation involves analyzing, synthesizing, reasoning, and judging based on perception. All decision-making plans and ideas are the results of mental processing (Del Missier et al., 2015). Ideation-based explanations are independent of systems and tactics to generate ideas, such as leverage points as generation seeds. Strategically manipulating associative memory involves focusing on substructures (Barbot, 2018).

Strategic decisions are constantly based on social, political, and economic situations. It’s done by analysis, synthesis, comparison, abstraction, and generalization, according to social-psychological demands (Heidari and Ebrahimi, 2016). Analysis and prediction of variable components in strategic decision-making depend on ideation, and ideation cannot be separated from other elements that drive strategic decision-making (Griessenberger et al., 2012). Therefore, the following hypotheses are proposed in this research:

H3a: Ideation has a positive effect on scanning ability.

H3b: Ideation has a positive effect on interpretation ability.

H3c: Ideation has a positive effect on action ability.

### Affective factors

Affective factors are classified into two types: low-level, namely emotion, and high-level, or sentiment. Positive or negative emotions characterize people. Positive emotions inspire decision-makers to work hard and be entrepreneurial. In this scenario, strategic decision-making establishes a higher aim, and the tools to achieve it are stable and complete (Treffers et al., 2020). While strategic decision-making in a negative emotional state reduces the decision-making goal and its measurements (Shukla et al., 2019). In a catastrophe, we should be calm, positive, and sensible. Before significant decisions, we should restrict emotional reactions, establish a good group decision-making environment, and enhance cohesiveness (Zhang et al., 2015). When people are overexcited, the brain’s exciting points are concentrated in one area and the other regions temporarily lose contact. Logic is almost lost, which hinders strategic decision-making (Shukla et al., 2019).

H4a: Emotion has a positive effect on scanning ability.

H4b: Emotion has a positive effect on interpretation ability.

H4c: Emotion has a positive effect on action ability.

The sentiment is a sophisticated, rational, steady, high-level feeling that combines emotion, and ethics. It has a normative role in people’s behavior and is the inner incentive for heroic activity (Kauffmann et al., 2019). Sentiment influences decision-making as a high-level emotion and social conduct including strategic decision-making. Moral decision-makers assess the impact of their decisions on the group, others, and society, not only on themselves (Morente-Molinera et al., 2019). Novel-minded decision-makers can relinquish their interests, restrain their impulses, and focus on group unity and societal impact in entrepreneurial strategic decision-making that may lead to enhanced performance (Sun et al., 2021). Therefore, the following hypotheses are proposed in this research:

H5a: Sentiment has a positive effect on scanning ability.

H5b: Sentiment has a positive effect on interpretation ability.

H5c: Sentiment has a positive effect on action ability.

### The influence of strategic decision-making ability on entrepreneurial performance

The strategy formulation of entrepreneurial enterprises regards the external environment as an important part of the strategic decision-making process (Chen, 2022). The scientific understanding of the environment, the accurate understanding of the characteristics, and changing trends of the environment, and seeking the best entrepreneurial path are the key to the success of the enterprise (Zhou and Wu, 2018; Xi et al., 2019). Decision-makers with strong environmental scanning ability can collect more comprehensive internal and external related information about the enterprise, which is also conducive to interacting with external stakeholders, obtaining strategic information, and constantly reducing information bias through information supplement and correction (Hongjia et al., 2010; Wang and Xu, 2019). Policymakers with strong interpersonal abilities can have a scientific understanding of the external competition, and economic, financial, and legal environment (Sarker and Palit, 2014). Decision-makers with strong action ability can design more strategic decision-making plans. Provide a diverse perspective for policymakers to evaluate strategic programs. Through the discussion, the decision-makers can evaluate the technological innovation strategic solutions comprehensively and objectively and ensure that high-quality solutions are selected from the numerous technological innovation strategic solutions (Yuetong, 2022). Therefore, strong strategic decision-making ability can make a suitable outline for enterprises in development and innovation. It points out the development direction of enterprises, guides entrepreneurial activities, and supervises the performance of entrepreneurship and entrepreneurship for a long time. Therefore, the following hypotheses are proposed in this research:

H6a: Scanning ability has a positive effect on EP.

H6b: Interpretation ability has a positive effect on EP.

H6c: Action ability has a positive effect on EP.

**FIGURE 1:**
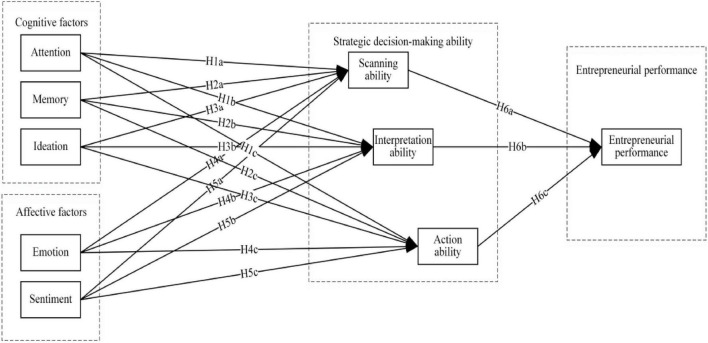
Conceptual model.

The conceptual model is presented here in Figure 1.

## Methodology

### Selection of the study methods

Structural Equation Modeling (SEM) has the advantage of simultaneously observing the relationship between variables and latent variables and the relationship between latent variables and latent variables and can also eliminate random measurement errors. Therefore, more accurate results can be obtained compared to the traditional regression analysis. Although the SEM method is widely used in empirical analysis research, only the linear relationship between the variables is considered in the analysis, which restricts its application depth. In order to make up for this defect of SEM, some scholars try to add interaction terms or quadratic terms to reflect the nonlinear relationship between variables (Tuu and Olsen, 2010), but the SEM model after adding interaction or quadratic terms is too complex, has poor computation, stability, and large parameter estimation is difficult disadvantages, making this method is difficult to be widely used in practice. Artificial Neural Network (ANN) can realize function approximation, data clustering, mode classification, optimization calculation, and other functions, and can automatically adjust the connection weight between the network nodes to fit the non-linear relationship of variables (Pasini, 2015). Applying ANN models can find complex linear and nonlinear associations between variables. Moreover, ANN models can perform more accurate predictions compared to linear analysis methods. However, the current topology structure of the current ANN model is mainly determined based on experience, and the neuronal nodes are often fully connected, which leads to the lack of a theoretical explanation of the path and degree of the influence between variables and neurons.

From the above analysis, we show that SEM is more flexible in reflecting causal relationships between variables than ANN but has limitations of difficulty to handle non-nonlinear relationships among variables. However, the ANN model can approximate the nonlinear relationship between the variables, and it poorly explains the input variable to influence the path and the degree of the output variable. To this end, we use a modeling approach combining SEM and ANN to test the series of hypotheses and theoretical models proposed in the paper. Structural equation-based model testing is performed using structural equation models as parameter estimation and hypothesis testing techniques to obtain the path of influence between variables. The SEM is based on the topology of the model and the structured ANN model according to the test results of the structural equation model.

### Analysis method of structural equation modeling

Structural Equation Modeling consists of a measurement model and a structural model. The measurement model analyses the link between an observed variable and a latent variable through Confirmatory Factor Analysis (CFA). Three matrices represent SEM.


(1) $$X=\Lambda \,x\,\xi +\sigma \,$$

(2) $$Y=\Lambda \,y\,\eta +\varepsilon \,$$

(3) $$\eta =B\,\eta +\Gamma \,\xi +\zeta \,$$

Equations (1, 2) are measurement models, *X* and *Y* represent observed variable vectors; Λx and Λy represent factor load matrix; ξ and η represent latent variable vectors; δ and ε represent measurement error vectors. Equation (3) is the structural model, *B* represents the “effect coefficient matrix of endogenous latent variables on endogenous latent variables,” Γ the “effect coefficient matrix of exogenous latent variables on endogenous latent variables,” and the residual items vector. Absolute and relative fitting indexes evaluate model fit. Model fit indexes are over 0.90, indicating that the data are well-fitted. The closer to 1, the better the model’s fit.

### Analysis method of structural equation modeling – artificial neural network

The SEM results determine the SEM-ANN topology. Widely utilized in the research of nonlinear issues, it may fit the relationship between variables by altering the weights of connections between network nodes (Foo et al., 2018; Shahzad et al., 2020). However, ANN topology is mostly determined by experience, and neurons are frequently connected. The model does not explain input and output variables, impact path, or degree between neurons. When ANN and SEM are integrated, SEM’s topology design determines the influence path of elements on strategic decision-making ability. ANN’s nonlinear mapping and self-learning abilities are utilized to fit the causal link among various elements. This overcomes linear and SEM parameter estimation difficulties and improves ANN’s topological structure. In this paper, SEM and ANN are used to model strategic decision-making ability.

Backpropagation (BP) neural networks are multi-layer feed-forward networks trained using error BP. BP trains the nodes’ connection weights. The BP algorithm separates forward and back propagation (Tan et al., 2014). Forwarding propagation transfers the input sample signal from the input layer to the output layer. The number of extraneous variables determines input layer nodes. The connection between networks is determined by measurement variables and potential variables, and node connection weight is generated by load and path coefficient (Shahzad et al., 2021). It calculates output error based on actual and expected output, distributes it to all layer nodes to obtain each node’s error signal, and uses this to alter weight. After the changes, forward propagation is reprocessed, and network output error is reduced. MSE and *R*^2^ measure algorithm accuracy. Less MSE means higher algorithm accuracy. *R*^2^ around 1 indicates a stronger model interpretation.

## Experiments

### Variables measurements and data source

The measures of attention typically refer back to work by Jugovac and Cavallero (2012) regarding attentional focus, focus stability, focus allocation, and focus transfer. As far as how memory is evaluated, most people look to studies conducted by Luber et al. (2013) on memory speed, memory stamina, and memory correctness. Specifically, the work of Campos-Blázquez et al. (2020) is cited as a primary source for determining how to quantify agility, flexibility, and depth of thought in the realm of ideation. In terms of tolerance, optimism, melancholy, and rage, the studies of Goldstein et al. (2013) are the most frequently cited when discussing the quantification of emotional states. Kazmaier and van Vuuren’s (2020) work on measuring morality, responsibility, and reason plays a significant role in the field. Scanning proficiency is evaluated based on how quickly, cheaply, and effectively it can gather information (Thomas et al., 2009).

According to Autier and Picq (2005) the ability to interpret information about one’s external environment, internal resources and capabilities, strengths and weaknesses, opportunities and threats, are the key factors in evaluating a person’s level of interpretive ability. The work of Ruigrok et al. (2006) is most often cited when discussing the many approaches, breadth of evaluations, and precision of selection available for gauging action ability. The measures of EP refer back to work by Baoshan et al. (2009), from the financial indicators and non-financial indicators of two aspects, regarding cash assets, return on investment, Interest rate, return on assets, and a number of new product items. This paper adopts the following two ways of getting data: the first is to obtain the help of local government departments, send the electronic questionnaire *via* e-mail and collect it by mail; the second is to fill in the paper questionnaire and collect it on the spot. A total of 1,500 questionnaires were sent out, 1,247 were returned, and 1,126 were considered for analysis after removing those with unengaged responses, missing data, and other issues. The significance level for the independent sample *t*-test was 0.498 (>0.05), indicating that there was no discernible difference between the three groups and that they could be combined.

### Empirical analysis based on structural equation modeling

Each variable’s reliability is tested using SPSS 21.0. The variables’ convergent validity, discriminant validity, and reliability were assessed using the SEM method in AMOS 24.0. Table 1 shows the findings.

**TABLE 1:** Test results of reliability and validity of variables.

Variables	Items	Loadings	Cronbach’s α	AVE	CR
Attention (AT)	AT1	0.81	0.84	0.56	0.84
	AT2	0.71			
	AT3	0.72			
	AT4	0.75			
Memory (ME)	ME1	0.82	0.84	0.64	0.84
	ME2	0.79			
	ME3	0.79			
Ideation (ID)	ID1	0.76	0.79	0.56	0.79
	ID2	0.74			
	ID3	0.75			
Emotion (EM)	EM1	0.79	0.86	0.60	0.86
	EM2	0.82			
	EM3	0.75			
	EM4	0.75			
Sentiment (SE)	SE1	0.78	0.83	0.61	0.83
	SE2	0.80			
	SE3	0.77			
Scanning ability (SC)	SC1	0.82	0.81	0.60	0.81
	SC2	0.74			
	SC3	0.74			
Interpretation	IN1	0.79	0.84	0.57	0.84
ability (IN)	IN2	0.70			
	IN3	0.79			
	IN4	0.74			
Action ability (AC)	AC1	0.76	0.81	0.59	0.81
	AC2	0.78			
	AC3	0.77			
Entrepreneurial	EP1	0.73	0.88	0.59	0.88
performance (EP)	EP2	0.78			
	EP3	0.79			
	EP4	0.77			
	EP5	0.77			

The reliability study revealed that each variable’s Cronbach’s value was larger than the standard of 0.70, indicating that the latent variables were reliable. The CFA of measurement model showed *x*^2^/*df* = 1.031, <2.0; RMSE = 0.012, less than 0.05; GFI = 0.924, CFI = 0.998, TLI = 0.997, all exceeding the specified critical value of 0.90, measuring the overall fitness of the model. The model’s values coincide with the sample data. Each measure’s factor loading was above 0.70, indicating high convergent validity. Each variable’s CR exceeds 0.70. Each variable’s AVE was above 0.50, indicating high discriminant validity. The variable measurement model has a good validity structure in general and can be investigated further.

The AMOS 24.0 examines the SEM of the enterprise’s sample data and the structural model’s overall fitting degree using the SEM analysis method to test the conceptual model. From the absolute fitting index, we can see that *x^2^/df* = 1.074, Less than 3 to achieve significance; RMSEA = 0.008. The model presentation is satisfactory at this stage. All three measures of relative fit (NFI, GFI, and CFI) are higher than the theoretical requirement of 0.90. All the results from the statistical analysis were statistically significant. This supports the validity of the model.

**FIGURE 2:**
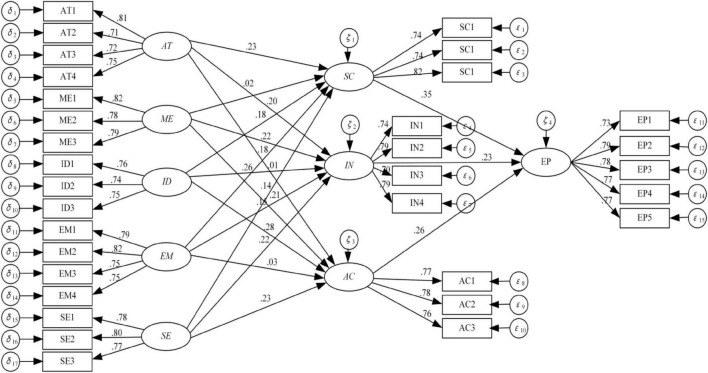
The fitting results based on structural equation modeling (SEM).

Examining the path coefficient’s significance is a primary way that SEM helps researchers confirm their research hypotheses. Coefficients with positive signs indicate a significant positive relationship between the two variables. The hypothetical relationship does not true if the coefficient is not significant. This hypothetical relationship, as seen in Figure 2 and Table 2, is briefly discussed below.

**TABLE 2:** Hypothesis test results.

Variable relation	Path coefficient	*P*-value	Hypothesis	Verification result
AT—>SC	0.23	***	H1a	Supported
AT—>IN	0.20	***	H1b	Supported
AT—>AC	0.01	0.978	H1c	Not supported
ME—>SC	0.02	0.482	H2a	Not supported
ME—>IN	0.22	***	H2b	Supported
ME—>AC	0.21	**	H2c	Supported
ID—>SC	0.19	0.398	H3a	Not supported
ID—>IN	0.26	***	H3b	Supported
ID—>AC	0.28	***	H3c	Supported
EM—>SC	0.18	**	H4a	Supported
EM—>IN	0.15	0.259	H4b	Not supported
EM—>AC	0.03	0.356	H4c	Not supported
SE—>SC	0.14	0.458	H5a	Not supported
SE—>IN	0.22	*	H5b	Supported
SE—>AC	0.23	***	H5c	Supported
SC—>EP	0.35	***	H6a	Supported
IN—>EP	0.23	**	H6b	Supported
AC—>EP	0.26	***	H6c	Supported

***, **, and * indicate that they have passed the test at 1, 5, and 10% significance, respectively.

**FIGURE 3:**
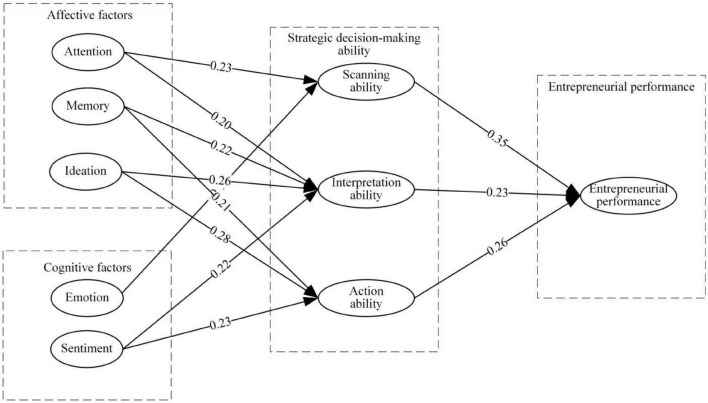
Influencing factors model of strategic decision-making capacity based on structural equation modeling (SEM).

As depicted in Figure 3, a model of influencing elements of strategic decision-making ability based on SEM has been constructed by removing the hypothetical path that was rejected following the results of the verification.

From the perspective of the relationship between cognitive factors and decision-making ability, the structural equation model results show that attention has no significant influence on action ability, memory on scanning ability, and thinking on scanning ability, which does not accord with the assumptions of this paper. The reason may be that some of the cognitive factors have not been effectively played because the strategic decision-makers make decisions.

From the relationship between emotional factors and decision-making ability, the structural equation model structure shows that emotion has no significant influence on paraphrasing ability, emotion on action ability, and emotion on scanning ability, which does not accord with the assumptions of this paper. May emotions have a negative impact on strategic decisions, as positive emotions make people optimistic or pessimistic about risk assessment of decision, strategic decision quality has a positive and negative effect, positive effect helps to improve the strategic decision quality, and vice versa. These two effects offset the board of social capital on technology innovation strategic decision quality influence is not significant.

### Empirical analysis based on structural equation modeling – artificial neural network

**FIGURE 4:**
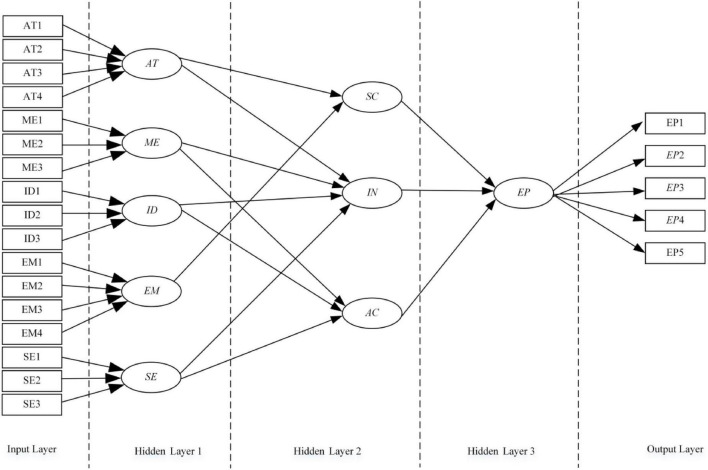
A structured neural network model structure.

Figure 4 shows a structured neural network model based on SEM results. In a structured neural network, five exogenous latent variables provide 17 inputs, while one endogenous latent variable generate five outputs. The first hidden layer represents an external latent variable, and there are five nodes. The second hidden layer represents the endogenous latent variable, three nodes. The third hidden layer represents the endogenous latent variable, one node.

**FIGURE 5:**
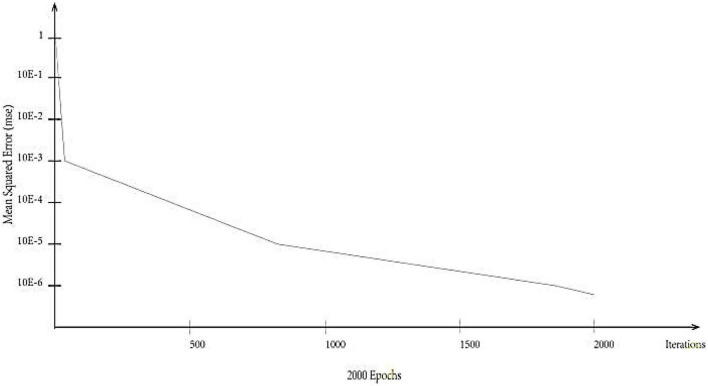
Neural network test results.

Using MATLAB neural network training model parameters, 788 training samples and 338 testing samples are used. Input, hidden, and output layers pick logistic. Maximum learning iterations (network steps) can be set to 2,000, target accuracy to 0.00, and learning efficiency to 0.1. MSE and R2t are determined using the trained model on test data. The RMSE of each test index is 0.1, and the maximum error is 0.2 (Figure 5). The model converges better after 2,000 iterations. Each measurement index’s *R*^2^ is over 0.5, suggesting acceptable model fitting.

**FIGURE 6:**
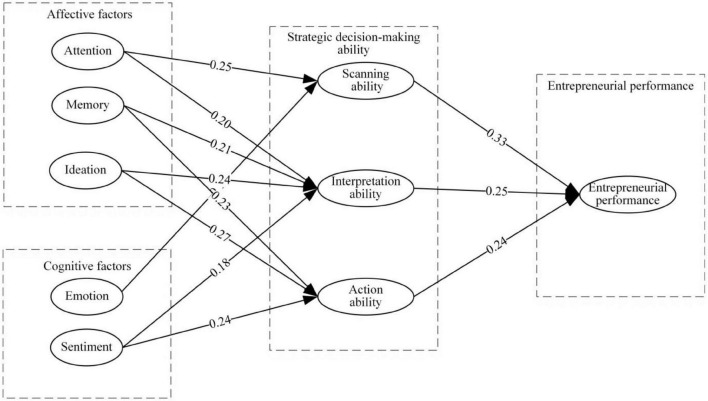
Influencing factors model of strategic decision-making capacity based on structural equation modeling – artificial neural network (SEM-ANN).

Normalized neuronal connection weight reflects each factor’s influence. A neural network study reveals that various factors affect strategic decision-making in distinct ways. For scanning ability, attentiveness is 0.25 and emotion is 0.19. Ideation has the biggest weight (0.27), followed by memory and attention (0.21 and 0.20), and feeling (0.18). For action ability, ideation weighs 0.24, followed by memory, and sentiment. Strategic decision-making ability affect EP in distinct ways, scanning ability is 0.33, interpretation ability is 0.25, and action ability is 0.24 shown in Figure 6.

## Conclusion

This paper proposes an SEM-ANN-based method for measuring strategic decision-making ability, and empirical analysis proves its potential effectiveness. A structured neural network model can describe the link and influence between network nodes and increase interpret ability. ANN can illustrate nonlinear relationships between influencing elements in SEM. Neural network nonlinear fitting improves model fit. Attention, memory, ideation, emotion, and sentiment influence strategic decision-making. Five factors affect strategic decision-making. Learning to interact with others is important for personal growth and professional relationships. Entrepreneurs with strong cognitive abilities can build a large, high-quality social circle to promote their business and get the ultimate performance. This study has the following implications and we also provide the limitations and future research directions.

### Theoretical implications

This research found that entrepreneurs can benefit from enhancing the following facets of their strategic decision-making abilities. Prioritize attention enhancement because it has a sizeable effect on scanning speed and a sizeable favorable effect on interpretation speed. Entrepreneurs should train themselves to observe things methodically and deliberately, with a focus on noticing minute shifts and variances in the decision-making environment and the psychology of those involved. Second, cognition enhancement should be a priority. The capacity for remembering information greatly aids in understanding and acting. A better decision-maker is one who actively works to develop their memory, studies the art of memory, and places a premium on using the memory method. Third, think about how to better your ideas. Entrepreneurial ability to generate new ideas is the single most important aspect of their intelligence. They should be adept at letting their imaginations run wild, challenging conventional wisdom, and looking at issues from many angles. In the fourth place, try to consciously hold on to a positive feeling. If you want to avoid being swayed by your emotions when deciding, you should make your choice after the excitement has died down. To foster settings conducive to evidence-based decision-making, decision-makers must acquire the skills necessary to consciously nurture and cultivate noble sentiments. The government should allocate funds to improve the quality of required education and training.

### Practical implications

The research conclusion of the paper enrich and strengthen the evidence of the positive influencing factors of strategic ability, empirically prove the relationship between strategic decision-making ability and EP, and put forward practical suggestions for improving decision-making ability and EP. However, the following limitations remain, and the data cross-section design makes the study results have some common methodological bias. Although the CFA was passed, the relevant impact is still not completely excluded, and future studies can use a longitudinal design for further verification.

### Limitations and future directions

Apart from the implications, our study has a few limitations that could be covered by future researchers. First, this cross-sectional study used data from a single source, which may introduce methodological biases and causal constraints when assessing relationships. Single-source data are unsuitable for robust findings, even if we have tried to mitigate typical procedure bias and found no bias. To validate the model, we recommend collecting data on various time lags in future research. Second, the data is collected only for one country which may neglect the potential effect of cultural aspects. Therefore, it is also recommended that future scholars used cross-cultural data to analyze the EP by considering various cultural factors.

## Data availability statement

The raw data supporting the conclusions of this article will be made available by the authors, without undue reservation.

## Ethics statement

Ethical review and approval was not required for the study on human participants in accordance with the local legislation and institutional requirements. Written informed consent for participation was not required for this study in accordance with the national legislation and the institutional requirements.

## Author contributions

JF: conceptualization, methodology, data curation, and writing – original draft preparation. PH: supervision, fund acquisition, and project administration. WZ: software, formal analysis, and data curation. AK: validation and writing – review and editing. All authors equally contributed to revising and finalizing the manuscript.
